# Oculomotor Nerve Palsy Secondary to Posterior Communicating Artery Aneurysm: A Narrative Review and Proposed Treatment Algorithm

**DOI:** 10.31083/RN40930

**Published:** 2025-11-30

**Authors:** Yuanhong Ge, Qingjia Lai, Yunsen Zhang, Yao Wang, Xuejun Xu

**Affiliations:** ^1^Department of Neurosurgery, Chengdu Second People’s Hospital, 610017 Chengdu, Sichuan, China; ^2^Department of Rehabilitation, Care Alliance Rehabilitation Hospital of Chengdu, 610000 Chengdu, Sichuan, China

**Keywords:** clipping, endovascular treatment (EVT), oculomotor nerve palsy (ONP), posterior communicating artery aneurysm (PcomA), recovery, review, clipado, tratamiento endovascular (EVT), parálisis del nervio motor ocular común (ONP), aneurisma de la arteria comunicante posterior (PcomA), recuperación, revisión

## Abstract

**Background:**

Oculomotor nerve palsy (ONP) is a condition characterized by ptosis, restricted eye movement, and pupillary abnormalities, with causes ranging from congenital to acquired factors. Among these, posterior communicating artery aneurysm (PcomA) represents the most clinically urgent due to the risk of rupture. Despite its significance, no standardized treatment guidelines currently exist. This narrative review aims to summarize current treatment approaches and provide a decision-making framework for clinicians.

**Methods:**

A literature review was conducted using Web of Science and PubMed from inception to December 30, 2024, with additional sources identified via manual reference searches.

**Results:**

Both aneurysm clipping and endovascular therapy are effective for treating PcomA-induced ONP. Endovascular techniques include coil embolization, stent- or balloon-assisted coiling, flow diverter placement, and intrasaccular flow disruption device placement. Surgical clipping is preferred in younger patients (under 60 years old), those with ONP symptoms longer than 7 days, an aneurysm size ≥7 mm, or complete ONP. In contrast, endovascular therapy is recommended for older patients, those in poor health, or undergoing treatment with antithrombotic agents. Emerging evidence suggests flow diverter placement is a promising direction, though further research is warranted.

**Conclusion:**

This review proposes a therapeutic algorithm to aid in clinical decision-making. The choice between aneurysm clipping and endovascular therapy should be individualized, taking into account patient-specific clinical factors.

## Abstract (es)

**Antecedentes:**

La parálisis del nervio motor ocular común (ONP, oculomotor nerve palsy) es una afección caracterizada por la ptosis, el movimiento ocular restringido y anomalías pupilares, cuyas causas pueden ser tanto congénitas como adquiridas. Entre ellas, el aneurisma de la arteria comunicante posterior (PcomA, Posterior communicating artery Aneurysm) representa la urgencia clínica más grave debido al riesgo de rotura. A pesar de su importancia, actualmente no existen directrices de tratamiento estandarizadas. Esta revisión narrativa tiene como objetivo resumir los enfoques terapéuticos actuales y proporcionar un marco de referencia para la toma de decisiones por parte de los médicos.

**Métodos:**

Se realizó una revisión bibliográfica utilizando Web of Science y PubMed desde sus inicios hasta el 30 de diciembre de 2024, y se identificaron otras fuentes mediante búsquedas manuales de referencias.

**Resultados:**

Tanto el clipado del aneurisma como la terapia endovascular son eficaces para tratar la ONP inducida por PcomA. Las técnicas endovasculares son la embolización con espirales, la embolización asistida por stent o balón, la colocación de un desviador de flujo y la colocación de un dispositivo de interrupción del flujo intrasacular. Se prefiere el clipado quirúrgico en pacientes más jóvenes (menores de 60 años), quienes tienen síntomas de ONP durante más de 7 días, un tamaño de aneurisma ≥7 mm u ONP completa. Por el contrario, se recomienda la terapia endovascular para pacientes de edad avanzada, quienes no tienen buena salud o que están en tratamiento con agentes antitrombóticos. Las pruebas emergentes sugieren que la colocación de un desviador de flujo es una opción prometedora, aunque se precisa más investigación.

**Conclusión:**

Esta revisión propone un algoritmo terapéutico para ayudar en la toma de decisiones clínicas. La elección entre el clipado del aneurisma y la terapia endovascular debe ser individualizada, teniendo en cuenta los factores clínicos específicos de cada paciente.

## 1. Introduction

Oculomotor nerve palsy (ONP) encompasses a range of conditions resulting in ptosis, restricted eye movement, dilated pupils, and sluggish or absent light reflexes, and can be classified into congenital and acquired forms [1]. Acquired ONP can stem from microvascular issues, trauma, tumors, aneurysms, and other causes. Among these causes, intracranial aneurysms pose the greatest danger, as ONP caused by such aneurysms may herald an impending rupture [2, 3, 4]. The mortality and disability rates are alarmingly high following aneurysm rupture, with pre-hospital mortality rates reaching 22%–26% and in-hospital mortality rates as high as 19%–20% [5]. Given that the oculomotor nerve in the subarachnoid space of the basal cisterns is in close proximity to the posterior communicating artery aneurysm (PcomA) arising from internal carotid artery, the majority of ONP-causing aneurysms are PcomAs (over 80%) [6]. Therefore, when ONP is detected, PcomA should be ruled out first. The optimal treatment strategy for ONP secondary to PcomA remains a subject of debate. To date, comparative studies of treatments have primarily been retrospective, lacking high-quality randomized controlled trials and consensus or guideline recommendations [7]. The purpose of this study is to provide a narrative review of ONP secondary to PcomAs, to assist with clinical decision-making.

## 2. Methods

We conducted a comprehensive search of PubMed and Web of Science from inception through December 30, 2024. The search terms were as follows: (“aneurysm*”) AND (“posterior communicating artery” OR “Pcom*”) AND (“oculomotor nerve palsy” OR “ONP” OR “third nerve palsy” OR “3rd nerve Palsy” OR “oculomotor nerve Disease” OR “oculomotor nerve paralysis”). Additional references were identified via manual citation. Eligible studies included human research (retrospective/prospective cohorts, case series, comparative studies, meta-analyses, or high-quality narrative reviews) with full-text availability in English or with English translations, specifically addressing ONP secondary to PcomA. Exclusions comprised ONP unrelated to PcomA (e.g., diabetes, trauma, tumor, other vascular causes), animal studies, abstracts/conference proceedings. This study is a narrative review and does not involve any primary data collection. Therefore, ethical approval and review by an Institutional Review Board (IRB) are not applicable.

## 3. Epidemiology

The annual incidence of acquired ONP in the general population is 3.3–4.7 per 100,000 [1, 8]. PcomAs are one of the causes of ONP. Due to anatomical features, 20%–32% of PcomAs may contribute to ONP [9, 10]. Early studies indicated that the most common cause of acquired ONP was PcomA, accounting for approximately 20–30% [11, 12, 13]. However, recent large-scale, population-based epidemiological studies have shown that ischemic cerebrovascular disease, rather than PcomA, is the most common cause of ONP [1, 14]. Among all causes of acquired ONP, aneurysms account for 6%, while in individuals over 60, aneurysm-related ONP makes up about 11% [1]. Notably, ONP caused by ischemic cerebrovascular disease rarely involves the pupil, whereas PcomA-induced ONP typically does [15]. Even though aneurysms may not be the most common cause of ONP, they remain the most dangerous. Therefore, when ONP is detected, PcomA should be ruled out first, especially in cases with pupillary involvement.

PcomA-induced ONP is most prevalent among individuals aged 40–60, similar to the age range for intracranial aneurysms [15, 16, 17]. ONP secondary to PcomA appears to be more common in women, which may be related to the higher incidence of intracranial aneurysms in females (relative risk of 1.3) [5, 9, 15, 16, 18]. However, two multicenter, large-sample studies showed that women accounted for approximately 84% of PcomA-induced ONP cases, a proportion that seems to exceed the percentage of women among all intracranial aneurysm patients [9, 16]. This may suggest that in PcomA cases, women have a higher incidence of ONP.

## 4. Anatomical Characteristics

**Fig. 1.**
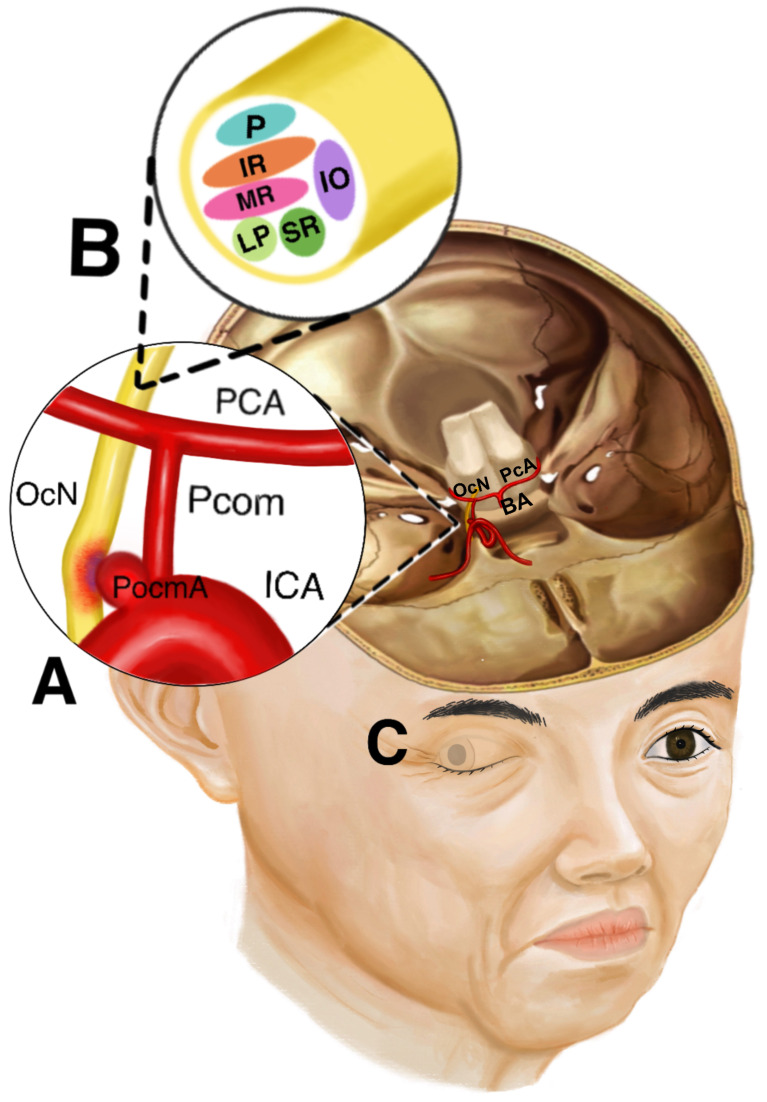
**Anatomy and Manifestations of Oculomotor Nerve Palsy Secondary to Posterior Communicating Artery Aneurysm**. (A) shows the anatomical relationship between the posterior communicating artery, the posterior communicating artery aneurysm, and the oculomotor nerve. (B) shows the topographical arrangement of the oculomotor nerve fibers. (C) shows the eyelid ptosis, the eye in an abducted position, and the dilated pupil due to oculomotor nerve palsy. PCA, posterior cerebral artery; ICA, internal carotid artery; Pcom, posterior communicating artery; OcN, oculomotor nerve; PcomA, posterior communicating artery aneurysm; P, pupillomotor fibers; IR, inferior rectus muscle; SR, superior rectus muscle; IO, inferior oblique muscle; MR, medial rectus muscle; LP, levator palpebrae superioris muscle; BA, basilar artery.

The oculomotor nerve, also known as the third cranial nerve, is anatomically divided into five segments following its emergence from the brainstem: cisternal, petroclinoid, cavernous, fissural, and orbital segments [19]. Damage to any part of this pathway can lead to ONP. The cisternal segment refers to the portion of the oculomotor nerve from its origin at the midbrain to its entry into the cavernous sinus, lying close to the edge of the tentorium cerebelli. This segment is approximately 15 mm in length and 2.5 mm in diameter [19, 20]. It is adjacent to the junction of the internal carotid artery and the posterior communicating artery, running slightly below and parallel to the posterior communicating artery, with a distance of about 1.7 mm between them. Therefore, PcomA is prone to causing ONP (Fig. 1).

The oculomotor nerve is commonly regarded as a purely motor nerve, primarily composed of somatic motor fibers and parasympathetic fibers [20]. However, few realize that it also contains a small number of sensory fibers [21, 22]. Motor fibers mainly innervate the levator palpebrae superioris (lifting the upper eyelid), superior rectus (moving the eyeball upward), inferior rectus (moving the eyeball downward), medial rectus (moving the eyeball inward), and inferior oblique (moving the eyeball inward and upward). Damage to these fibers results in ptosis and restricted eye movement. Parasympathetic fibers primarily innervate the pupillary sphincter (constricting the pupil) and the ciliary muscle (involved in the accommodation reflex). The sympathetic nervous system, which innervates the pupil dilator muscle (causing pupil dilation), consists of three levels of neurons: the first level is located in the posterolateral hypothalamus, sending fibers through the brainstem to the second level (C8-T1), which then sends fibers to the third level (the superior cervical ganglion located in front of the second and third cervical vertebrae) [23, 24]. Thus, the contraction state of the pupillary sphincter and the pupil dilator muscle determines the size of the pupil. When the parasympathetic fibers of the oculomotor nerve are damaged, the pupillary sphincter loses its antagonistic effect on the pupil dilator muscle, causing the pupil to dilate. Conversely, when the cervical sympathetic nerve is damaged, the pupil constricts (as seen in the well-known Horner’s syndrome). In a cross-section of the oculomotor nerve, parasympathetic fibers are located in the superficial layer of the nerve trunk [24, 25]. Therefore, when the oculomotor nerve is compressed, pupil changes are theoretically the first to occur. However, if the aneurysm compresses the oculomotor nerve from below, it may result in pupil-sparing ONP, which is rare [6, 26, 27]. This is because the fibers innervating the pupil are located in the superior-medial superficial layer of the oculomotor nerve [27, 28]. Autopsy and animal studies have shown that the oculomotor nerve contains a small number of sensory fibers (originating from the ophthalmic division of the trigeminal nerve), which are involved in the perception of eye position and periorbital pain [21, 22].

## 5. Presentation

ONP secondary to unruptured PcomA typically manifests as isolated oculomotor nerve palsy, characterized by the absence of other neurological deficits except for headache or periorbital pain [14]. Depending on the affected muscles, ONP can be classified into extraocular muscle palsy (ptosis or restricted eye movement) and intraocular muscle palsy (pupillary involvement). If PcomA-related ONP is accompanied by PcomA rupture, symptoms associated with subarachnoid hemorrhage (SAH) such as headache, nausea, vomiting, increased intracranial pressure, or altered consciousness, motor, and sensory dysfunction, and seizures may also be present.

Based on the degree of extraocular and intraocular muscle palsy, ONP can be categorized into partial and complete forms (Table 1, Ref. [28]).

**Table 1.** **The degree of partial and complete ONP [28]**.

ONP degree		Description
Extraocular muscle	Normal function	Full range of motion in all directions with no ptosis.
	Partial dysfunction	Ptosis greater than 2 mm, reduced range of motion in appropriate directions with or without eye deviation in the primary position, or a combination of these.
	Complete dysfunction	Eye deviated downward and outward in the primary position with no movement in appropriate directions.
Intraocular muscle	Normal Function	Pupils equal in size and reaction; anisocoria less than 1.0 mm is considered normal only if pupils react equally in room light.
	Partial dysfunction	Pupil dilated, difference ≥1.0 mm compared to the other side (regardless of reaction) in room light; or pupil diameter difference <1 mm but with abnormal reaction.
	Complete dysfunction	Pupil dilated and fixed.

ONP, oculomotor nerve palsy.

Ptosis results from dysfunction of motor fibers supplying the levator palpebrae superioris. Restricted eye movement (inward, upward, and downward) results from involvement of the superior rectus, inferior rectus, medial rectus, and inferior oblique muscles, resulting in a characteristic abducted and depressed eye position. Patients with extraocular muscle palsy may experience difficulty opening the affected eye and diplopia. In the vast majority of PcomA-induced ONP cases (approximately 98.6%), the pupil is affected, typically presenting with dilated, poorly reactive pupils, although occasionally constricted or oval-shaped pupils may occur [6]. Early compression of the oculomotor nerve may cause irritation, explaining the rare occurrence of constricted pupils. A small percentage of PcomA-induced ONP cases do not involve the pupil [6, 29].

More than 50% of patients with unruptured PcomA and ONP experience ipsilateral periorbital pain [11, 21, 30, 31]. However, few studies have focused on the cause of this pain. The exact mechanism remains unclear, but three hypotheses may explain it:

(1) Local dural irritation: The tentorium cerebelli has recurrent branches from the ophthalmic division of the trigeminal nerve. PcomA may irritate the adjacent tentorium, causing referred pain in the periorbital area. This hypothesis is well supported by the fact that meningeal irritation tests in the corresponding region can induce pain in the periorbital area [32, 33].

(2) Trigeminal sensory modulation disorder: The oculomotor nerve contains a small number of sensory fibers that connect with the ophthalmic division of the trigeminal nerve and inhibit trigeminal afferent information [21, 22].

(3) Extraocular muscle fatigue: Restricted eye movement due to ONP leads to muscle fatigue and local lactic acid accumulation, which is transmitted to the central nervous system via the ophthalmic division of the trigeminal nerve, causing a feeling of eye strain. The common experience of eye strain after prolonged use of the eyes (e.g., reading or watching TV) seems to support this view.

## 6. Diagnosis

Digital subtraction angiography (DSA) has long been considered the gold standard for diagnosing intracranial aneurysms. However, DSA is invasive, with an estimated risk of neurological and systemic complications ranging from 1% to 2%, which is higher in the elderly or those with cerebral arteriosclerosis [28, 34]. In the past, to reduce unnecessary DSA procedures, some scholars developed classification systems for ONP to identify the patients at high risk for PcomAs and confirm the diagnosis through DSA. These classification systems were based on the degree of ONP, pupillary involvement, age, and other clinical features [28, 35]. However, these systems were not always reliable [34]. With the advancement of imaging technology, computed tomography angiography (CTA) or magnetic resonance angiography (MRA) can now identify the vast majority of intracranial aneurysms, especially those larger than 3 mm [36, 37]. According to the literature, the smallest PcomA causing ONP is at least 4 mm in size, which can be detected by current noninvasive imaging modalities such as CTA or MRA [36, 38, 39]. The older ONP-based risk stratification tool has relatively low sensitivity and specificity, and it also lacks external validation. It is estimated that the misdiagnosis rate of PcomA-induced ONP, based on the ONP classification system, is no less than 10%. Considering the high disability and mortality rates associated with aneurysm rupture, this misdiagnosis rate needs to be significantly reduced. Currently, non-invasive MRA or CTA can almost confirm the diagnosis of ONP-causing PcomA with nearly 100% accuracy [34, 36]. Given the advantages of CTA and MRA (non-invasive, low cost, minimal radiation, simplicity, and high specificity and sensitivity for aneurysm diagnosis), the applicability of ONP classification systems has diminished [34, 39]. CTA and MRA can also help differentiate ONP secondary to other reasons, such as tumors or strokes. Therefore, all patients with ONP should be advised to undergo CTA or MRA as soon as possible to quickly determine the cause of ONP.

## 7. Treatment

### 7.1 Etiological Treatment

For ONP induced by PcomA, the primary focus is on managing the PcomA. This not only prevents the catastrophic consequences of aneurysm rupture but also alleviates the compression on the oculomotor nerve, thereby improving ONP. The evolution of treatment strategies for PcomAs reflects a transition from an initial emphasis on surgical treatment (1987–1995) to a period of peak utilization of both surgical and endovascular therapy (EVT) (2005–2013), and more recently, a focus on innovative devices and comparative studies (2014–2022) [40].

#### 7.1.1 Surgical Treatment

The surgical treatment of intracranial aneurysms has a history of nearly 300 years, evolving through techniques such as carotid artery occlusion, aneurysm wrapping, and precise clipping of the aneurysm neck [41]. Currently, precise clipping of the aneurysm neck is the classic method for surgical treatment of intracranial aneurysms. After clipping of a PcomA, the compression on the oculomotor nerve is relieved. According to the literature, the overall efficacy rate (complete recovery + partial recovery) of ONP after clipping treatment for PcomA is estimated to be between 68.2% and 100% [9, 10, 42, 43, 44].

PcomA clipping itself may also induce ONP. The reported incidence of surgery-induced ONP is 4.4% [45]. Risk factors for induction include large aneurysms (greater than 10 mm), intraoperative aneurysm rupture, and a time interval of more than 14 days between aneurysm bleeding and surgery [45]. These factors may be related to direct damage to the oculomotor nerve during surgical dissection, injury to the feeding artery, or the space-occupying effect of the aneurysm clip.

#### 7.1.2 Endovascular Treatment

Since the introduction of detachable bare platinum coils (Guglielmi) in 1990, the use of coils for EVT in treating intracranial aneurysms has gained widespread acceptance [46]. Since then, EVT for intracranial aneurysms has become increasingly popular. Currently, EVT methods include simple coil embolization, stent-assisted coiling (SAC), balloon-assisted coiling (BAC), flow diverter (FD) placement, and intrasaccular flow disruption device placement. According to the literature, the overall efficacy rate (complete recovery + partial recovery) of ONP after EVT for PcomA-related ONP is estimated to be between 61.3% and 100% [9, 10, 16, 42, 43, 44, 47]. FD has become a hot topic in the treatment of intracranial aneurysms in recent years. The use of FD alone or in combination with loose coil packing does not increase the space-occupying effect and can reduce the size and pulsatility of the aneurysm, thereby improving PcomA-related ONP [48, 49, 50]. However, the incidence of stroke complications associated with FD is relatively higher. As reported by Boulouis G [49], for patients with intracranial aneurysms presenting with compressive neuro-ophthalmological symptoms undergoing FD treatment, the overall complication rate is about 20%, with symptomatic ischemic stroke occurring in approximately 12.7% of cases and hemorrhagic stroke in around 5.5%.

#### 7.1.3 Which is the Best Strategy?

There has always been controversy over whether clipping or EVT is the preferred treatment for PcomA-related ONP. Some studies have shown that clipping is superior to EVT [15, 17, 18, 51], while others have found no significant difference between the two [27, 42, 52, 53]. Hall *et al*. [54] showed that for PcomAs <7 mm, there was no difference in ONP treatment efficacy between coiling and clipping. However, for PcomAs ≥7 mm, clipping was superior to coiling. A systematic review indicated that patients with ruptured PcomA and ONP benefit the most from clipping [15]. Another systematic review indicates that within 12 months after procedure, the recovery rate of ONP following clipping is superior to that of coiling. However, after 12 months, the difference between clipping and coiling is not significant [55]. Overall, both clipping and coiling have similar and relatively low complication rates [10, 43, 44]. The comparison of outcomes between clipping and EVT is summarized in Table 2 (Ref. [43, 44, 55]).

**Table 2.** **The comparison of outcomes between clipping and endovascular treatment [43, 44, 55]**.

		Clipping	Endovascular Treatment
Time to recovery (mean ± standard deviation, days)		141.9 ± 120.3	128.3 ± 10.94
Overall recovery rate		68.2%–100%	61.3%–100%
Recovery rates at different follow-up time points			
	1 month	53%	17%
	3 months	69%	33%
	6 months	79%	48%
	12 months	90%	64%
	18 months	87%	64%
	24 months	86%	72%
Complications		5.6%	4.3%

**Fig. 2.**
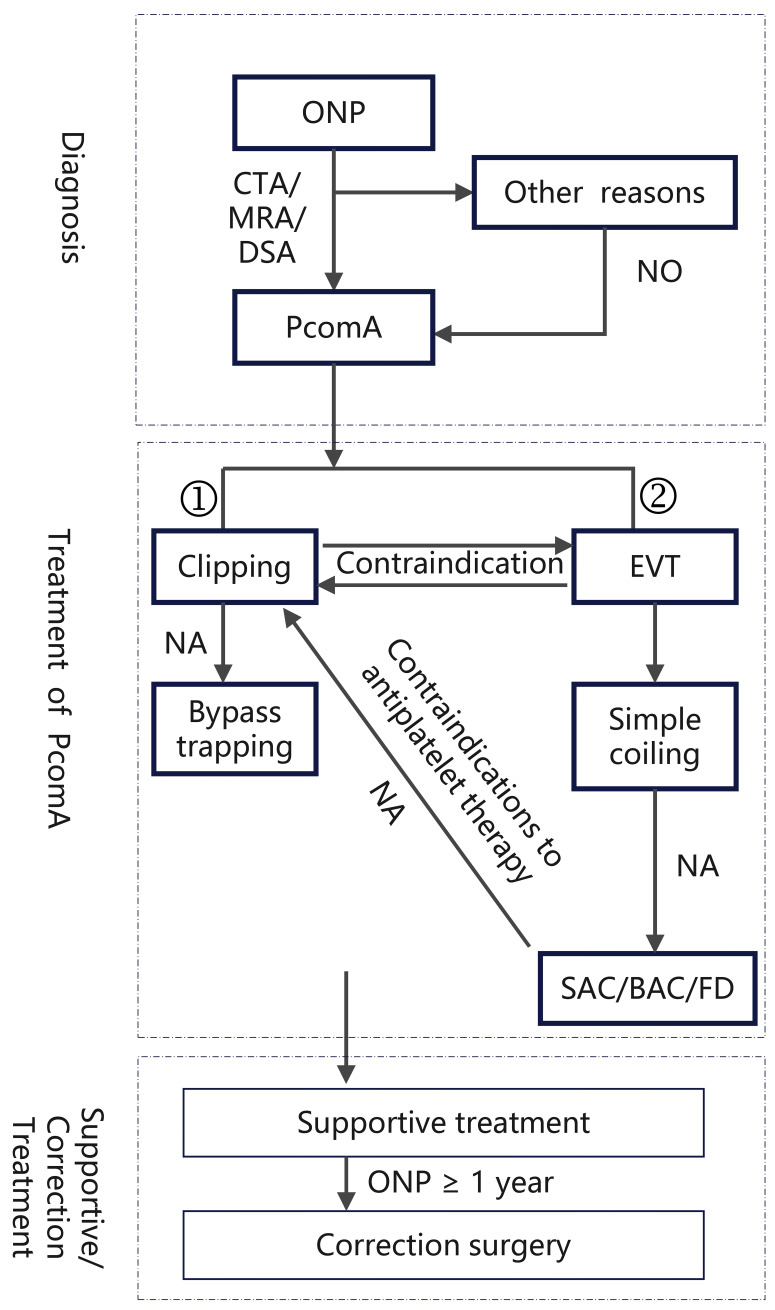
**Flowchart for the Diagnosis and Treatment of ONP Secondary to PcomA**. ①: This branch includes patients who are <60 years old, with ONP symptoms >7 days, aneurysm size ≥7 mm, ruptured aneurysm with large hematoma, complete ONP, or when EVT is not feasible (e.g., difficult access). ②: This branch includes patients who are ≥60 years old, with ONP symptoms ≤7 days, aneurysm size <7 mm, partial ONP, poor overall health, or ongoing/required antithrombotic therapy (anticoagulation or antiplatelet). CTA, computed tomography angiography; MRA, magnetic resonance angiography; DSA, digital subtraction angiography; PcomA, posterior communicating artery aneurysm; EVT, endovascular treatment; NA, not applicable; SAC, stent-assisted coiling; BAC, balloon-assisted coiling; FD, flow diversion.

It is difficult to determine which treatment method is the best, as there is currently a lack of prospective randomized controlled trials. Based on existing evidence, clipping seems to be more favorable for ONP recovery [15, 17, 18, 51]. However, older patients tend to be more inclined towards EVT [43, 44, 56]. In clinical practice, the choice of treatment strategy needs to take into account a variety of factors, such as (1) patient and physician preferences; (2) patient age and comorbidities: (3) the size and shape of the aneurysm. In any case, both EVT and clipping are effective. Therefore, we propose a diagnostic and therapeutic algorithm to aid in clinical decision-making (Fig. 2).

Among the different EVT methods, there is a lack of comparative studies on which is better for relieving ONP. Wang *et al*. [16] showed no difference in ONP recovery between simple coil embolization and SAC. For ONP secondary to intracranial aneurysms treated with FD, the relief rate within six months was 62.5% (10/16), and the relief rate within three years was 100% (13/13).

### 7.2 Supportive Treatment

Research on supportive treatments for ONP remains limited. Current strategies primarily encompass corticosteroids, methylcobaminate/vitamin B12, acupuncture, and rehabilitation therapies (such as electrical stimulation and vision interventions) [57, 58, 59, 60]. These measures may serve as adjunctive therapies in managing PcomA-related ONP.

### 7.3 Extraocular Muscle Correction Surgery

When symptoms of ONP persist for 12 months and affect work and daily life, extraocular muscle correction surgery may be considered [61, 62]. A systematic review analysis revealed that for ONP secondary to PcomA, the probability of ONP recovery gradually increases within the first 12 months following aneurysm treatment. However, beyond 12 months, this probability tends to stabilize [55]. Because the extraocular muscle correction surgery is typically carried out by ophthalmologists, it is not discussed in this review.

## 8. Prognosis

More than two-thirds of patients with PcomA-associated ONP exhibit improvement following treatment [9, 10, 42, 43, 44]. The median time for ONP recovery is approximately 2–3 months after treatment, with the fastest recovery occurring within days. However, if there is no complete recovery after three months, the likelihood of complete recovery is very low [9, 43, 44, 63]. Stiebel-Kalish *et al*. [64] categorized the degree of incomplete ONP recovery into three levels: (1) mild (mild upgaze deficit only when looking up), (2) moderate (upgaze and downgaze deficits when looking up, but no fixation deficit), and (3) severe (residual diplopia, mild adduction deficit, and mild upgaze deficit in primary gaze).

The order of recovery of extraocular and intraocular muscle function after PcomA treatment is as follows: levator palpebrae superioris, medial rectus, inferior rectus, superior rectus, pupillary sphincter, and ciliary muscle. Patients with incomplete recovery often exhibit residual upgaze or downgaze deficits or pupillary abnormalities [65, 66, 67]. This order of muscle function recovery reflects the degree of compression and injury to the nerve fibers, as well as the arrangement and quantity of related nerve fibers within the oculomotor nerve. The fibers innervating the pupillary sphincter and ciliary muscle are fewer in number, with pupillary fibers being even scarcer, accounting for only 3% of parasympathetic fibers, and are located in the peripheral part of the oculomotor nerve [24]. Therefore, they may suffer more severe damage and recover more slowly. Some patients may still experience varying degrees of blurred vision despite resolution of extraocular muscle deficits, possibly due to residual ciliary muscle dysfunction and its impact on lens refractive power.

Some studies have explored the factors influencing ONP recovery. Regardless of the treatment method, it is generally believed that the duration and severity of ONP prior to treatment are significant factors [10, 16, 42, 43, 47, 49]. The longer the duration of ONP, the more severe the damage to the oculomotor nerve fibers or blood supply, which implies poorer recovery potential. Several studies have shown that patients who undergo surgical treatment within 14 days of ONP onset have a significantly higher probability of ONP recovery compared to those treated after 14 days [16, 47, 63]. Zhong *et al*. [43] found that patients treated more than 7 days after ONP onset had a significantly lower probability of ONP recovery compared to those treated within 7 days (OR 0.325, 95% CI 0.465 to 0.973). Compared to complete ONP, incomplete ONP has a higher probability of complete recovery and requires less time for recovery [16, 43, 47]. Two large-sample, multicenter studies showed that ruptured PcomAs are more conducive to ONP recovery than unruptured PcomAs [9, 44]. Other factors that may be detrimental to ONP recovery include smoking, advanced age, and recurrence of PcomA [9, 42, 47, 49].

## 9. Conclusion and Future Direction

PcomA represents one of the significant etiological factors for ONP, frequently associated with pupillary involvement and periorbital pain, and exhibits a higher prevalence in women. In terms of treatment, both aneurysm clipping and EVT can effectively improve ONP, although clipping may have an advantage in ONP recovery. Additionally, ONP recovery is influenced by various factors, which are closely tied to the duration and severity of ONP prior to treatment initiation. The therapeutic algorithm proposed in this review aids in clinical decision-making.

Future research should prioritize prospective randomized controlled trials to directly compare the long-term efficacy of surgical clipping versus EVT in improving ONP recovery, particularly for PcomAs of varying sizes and morphologies. FD holds significant promise for treating PcomA-related ONP due to their minimally invasive profile, absence of space-occupying effects, and capacity to reduce aneurysm size and pulsatility. However, current clinical data supporting their use remain limited, underscoring the need for robust multicenter studies to validate these benefits. Further investigations should focus on optimizing FD design and deployment strategies to minimize complications (e.g., thromboembolic events, delayed aneurysm rupture). Innovations such as bioactive coatings to minimize thrombogenicity and adaptive stent designs that conform to anatomical variations may improve both procedural safety and clinical outcomes. Additionally, exploring adjunctive therapies—including neuroprotective agents to mitigate nerve damage and targeted rehabilitation protocols to address residual deficits—may improve functional recovery, especially in cases with delayed intervention.
